# The First Report of Cutaneous Nocardia Concava Infection in the United States

**DOI:** 10.7759/cureus.4740

**Published:** 2019-05-23

**Authors:** Jerry W Sayre, Ansley Lorch, Mehak Gandhi, Robert Yancey, Pulkit Arora

**Affiliations:** 1 Family Medicine, North Florida Regional Medical Center, Gainesville, USA

**Keywords:** nocardia concava, cutaneous, nocardiasis, us, case, patient, infection, nocardiosis

## Abstract

This is a case report presenting a rare disease, cutaneous Nocardia concava infection. There are currently only five published case studies worldwide describing infections caused by N. concava, and all of them occurred in Far East Asian countries (Japan, China, and Korea). Three of the reported cases were cutaneous infection cases. This is the sixth case to be reported in the world and the first in the United States. A 75-year-old Caucasian female was admitted to a community hospital with recurring cutaneous lesions for four months. Biopsy demonstrated the presence of Nocardia concava, confirmed by DNA sequencing on the hps65 gene. Treatment was achieved with sulfamethoxazole-trimethoprim. This case report creates awareness for family physicians to consider that cutaneous nocardiosis may mimic the cutaneous lesions of sporotrichosis. If a patient fails multiple treatment regimens, advanced microbiological evaluation methods such as DNA sequencing should be considered.

## Introduction

Nocardia concava is an aerobic, gram-negative, filamentous-branched bacteria of the order Actinomycetales, which was recently discovered in 2005 [1]. The genus Nocardia is comprised of species that are known to be an unusual cause of a wide spectrum of clinical diseases in both humans and animals [2] and can cause severe opportunistic infections in immunocompromised patients with major known risk factors, such as patients with AIDS and chronic pulmonary disease or who had solid organ transplantation [3]. Depending on the site of the infection and species that cause the infection, survival rate approximates 50% [4]. The wide-spectrum of clinical manifestations and lack of histological data makes it difficult to diagnose [1]. There are currently only five published case studies worldwide describing infections caused by N. concava, and all of them occurred in Far East Asian countries (Japan, China, and Korea) [1, 5, 6]. Three of the reported cases were of cutaneous infection, and all occurred in Japan [1].

This case report aims to create awareness for family physicians to consider that cutaneous nocardiosis may mimic the cutaneous lesions of sporotrichosis. If a patient fails multiple treatment regimens, advanced microbiological evaluation methods such as DNA sequencing should be considered.

## Case presentation

Sociodemographics and medical history

The patient was a 75-year-old Caucasian female, a retired seamstress and a widow with a previous history of smoking who lived alone and enjoyed gardening as a hobby. She had multiple chronic diseases including diabetes mellitus (DM), hypertension, chronic kidney disease (Stage III), anemia, and coronary artery disease.

Four months prior the patient sought medical care for skin vesicles followed by ulcerations of her left arm and was treated with oral antibiotics. With non-resolution of the “skin inflammation” she subsequently saw her dermatologist who treated her sporotrichoid-patterned lesions with serial courses of ciprofloxacin then clindamycin with only mild benefit. Biopsies were negative for infectious etiologies or malignancies.

The patient completed two courses of antibiotics and two weeks after completion, she noticed worsening of the nodules over her left lateral arm and sought care in a community hospital emergency room.

Pertinent physical exam

**Figure 1:**
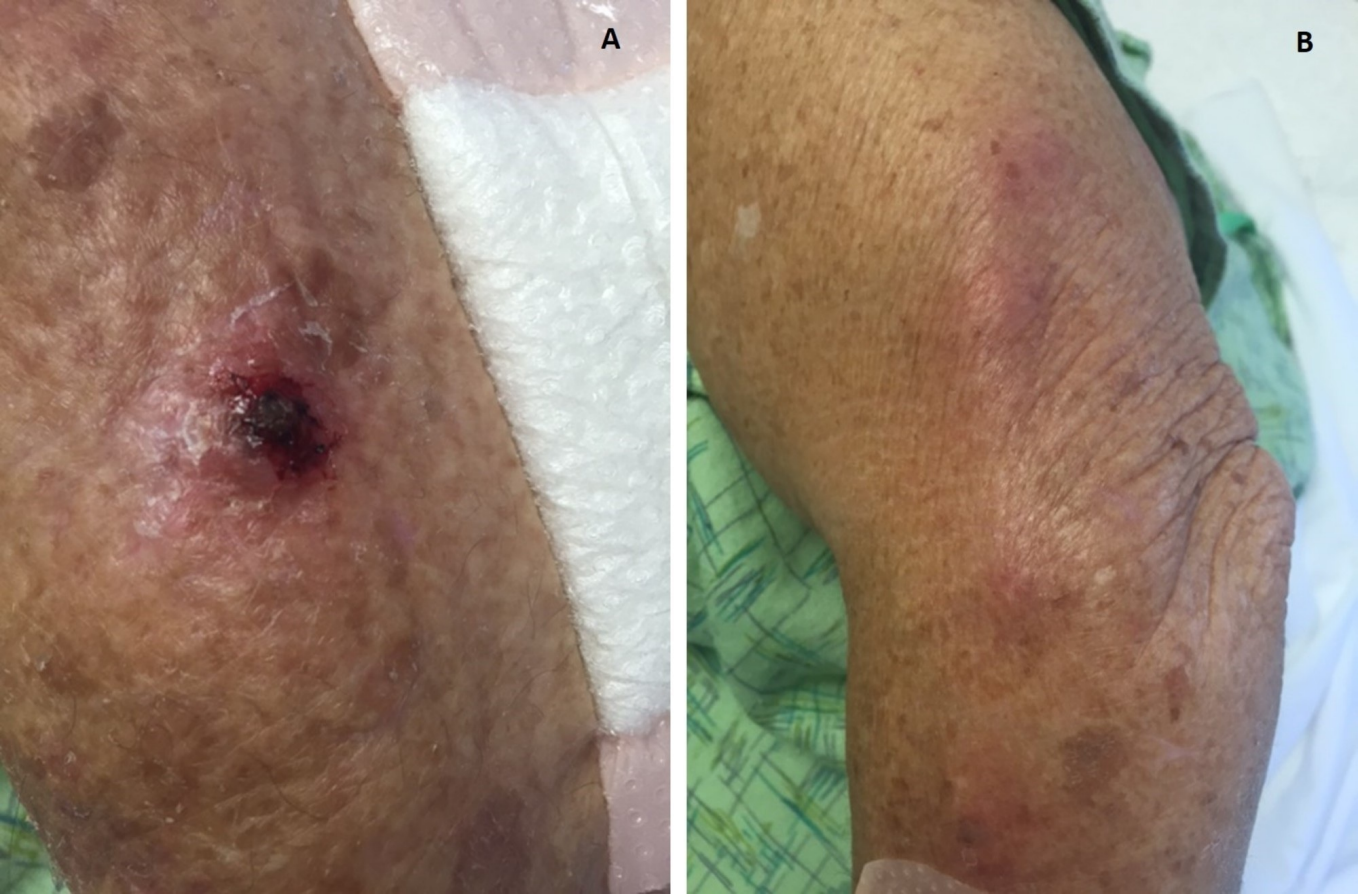
Photograph of the lesion resulting from cutaneous Nocardia concava infection; A) skin nodule B) erythema and swelling

The patient was afebrile and had stable vitals. Skin nodules were present along the superficial veins lateral to her left lateral epicondyle extending proximally and posteriorly around the triceps, in a characteristic spiral pattern. There were scattered areas of slight erythema and swelling, with crusting of approximately one centimeter in diameter. These lesions extended distally to the proximal dorsal lateral forearm with mild tenderness, erythema and warmth without crepitus or fluctuation (Figure 1). No lower extremity edema was observed and there was no suggestion of deep vein thrombosis.

Pertinent imaging/lab results

Left upper extremity Doppler ultrasound imaging was normal without evidence of deep vein thrombophlebitis. Initial blood biochemistry showed a WBC count of 13,000, which normalized by the next day. No electrolyte abnormalities were seen.

Management

The patient was initially given empiric parenteral vancomycin on admission. Infectious disease service was consulted. Due to the pattern of the nodules and history of gardening, there was concern for sporotrichosis. General surgery was consulted and a biopsy of the nodules was performed. The biopsy specimen was sent for extended culture. Presence of Nocardia concava on lesions was confirmed by DNA sequencing on the hps65 gene. Based on this finding, the patient was diagnosed with nocardiosis and treatment was initiated with sulfamethoxazole-trimethoprim.

## Discussion

In this patient with a primary cutaneous Nocardia concava infection, the initial physical exam revealed nodules presenting in a sporotrichoid pattern. This finding, along with the history of gardening, made us suspicious for sporotrichosis. Initial treatment with ciprofloxacin and clindamycin failed to eradicate the infection. DNA sequencing revealed infection of Nocardia concava.

There are several limitations to this study compounded by the lack of sufficient literature on Nocardia concava. Before the year 2000 in the United States, Nocardia species were identified primarily through standard bacteriological methods instead of gene sequencing. This diagnosis may be underreported because institutions do not have access to DNA sequencing methodology. Therefore, laboratory identification is challenging [3].

## Conclusions

This report aims to create awareness for family physicians to consider that cutaneous Nocardia may mimic the cutaneous lesions of sporotrichosis. If a patient fails multiple treatment regimens, advanced microbiological evaluation methods such as DNA sequencing should be considered.
